# Epidemiological Investigation and Genetic Analysis of Duck Circovirus in Korea from 2013 to 2022

**DOI:** 10.3390/ani14243630

**Published:** 2024-12-16

**Authors:** Cheng-Dong Yu, Sang-Won Kim, Cun-Xia Liu, Yue-Hua Gao, Yu-Feng Li, Jong-Yeol Park, Se-Yeoun Cha, Hyung-Kwan Jang, Min Kang, Bai Wei

**Affiliations:** 1 Department of Avian Diseases, College of Veterinary Medicine and Center for Avian Disease, Jeonbuk National University, Iksan 54596, Republic of Korea; yuchengdong@naver.com (C.-D.Y.); sangwonkim@jbnu.ac.kr (S.-W.K.); jyp0410@jbnu.ac.kr (J.-Y.P.); seyeouncha@jbnu.ac.kr (S.-Y.C.); hkjang@jbnu.ac.kr (H.-K.J.); 2 Institute of Poultry Science, Shandong Academy of Agricultural Sciences/Shandong Provincial Key Laboratory of Livestock and Poultry Breeding, Jinan 250100, China; liucunxia9@163.com (C.-X.L.); cwh143@163.com (Y.-H.G.); dicpd@163.com (Y.-F.L.); 3 Bio Disease Control (BIOD) Co., Ltd., Iksan 54596, Republic of Korea

**Keywords:** duck circovirus, epidemiological investigation, phylogenetic analysis, genetic diversity, recombination

## Abstract

Duck circovirus (DuCV) infections cause immunosuppression in ducks, potentially leading to significant economic losses for the duck farming industry. This study investigates the prevalence, genetic characteristics, and evolutionary trends of DuCV in Korea between 2013 and 2022. Samples from 184 farms across seven provinces were analyzed using polymerase chain reaction (PCR). The overall DuCV infection rate was 29.4% (54/184), with Jeollanam-do showing the highest prevalence (37.5%, 15/40). Ducks aged 3–6 weeks were most susceptible to infection, while ducklings younger than one week were rarely infected. Whole-genome sequencing was performed on 24 positive samples with phylogenetic analysis revealing that DuCV-1b is the predominant subtype in Korea (23/24 strains). Notably, a Korean DuCV-1a subtype strain was identified for the first time, showing close genetic relatedness to Chinese DuCV 1a strains. Novel subtype-specific amino acid variations in ORF1 and ORF2 were statistically analyzed and classified. Recombination analysis suggested some Korean DuCV-1b strains may have resulted from recombination events involving strains from different countries. This comprehensive study provides crucial insights into the current prevalence, genetic diversity, and evolutionary dynamics of DuCV in Korea, offering valuable data for developing effective control strategies and understanding the global epidemiology of this economically important avian pathogen.

## Simple Summary

Duck circovirus (DuCV) is a common DNA virus in ducks that can infect nearly all breeds of ducks. It is primarily transmitted via the cloacal–fecal–oral route, and ducks of all ages are susceptible. While DuCV infection alone is often subclinical, it can induce immunosuppression, which in turn increases the susceptibility to other common pathogens and exacerbates clinical symptoms. Currently, DuCV has a global distribution with a high infection rate, posing a serious threat to the duck industry. Recent mutations and the emergence of new genotypes, coupled with evidence suggesting that migratory birds may play a significant role in DuCV transmission, have heightened concerns about this virus. Therefore, this study conducted an epidemiological investigation of DuCV in samples collected from Korean farms between 2013 and 2022 and analyzed the genetic variation characteristics of DuCV strains during this period. These findings significantly enhance our understanding of the current epidemiological status of DuCV in South Korea and provide valuable data to support future control strategies.

## 1. Introduction

Duck circovirus (DuCV) is a non-enveloped, single-stranded circular DNA virus with icosahedral symmetry and a particle diameter of approximately 15–16 nm, making it the smallest known duck virus [1,2]. According to the latest report released by the International Committee on Taxonomy of Viruses (ICTV) in 2023, DuCV is a member of the *Circovirus* genus in the *Circoviridae* family (https://ictv.global/taxonomy; accessed on 14 August 2024). The DuCV genome sequence length ranges between 1755 and 1996 base pairs and contains three major open reading frames (ORFs) and two intergenic regions (IRs) [1,3,4]. ORF1 encodes a replication-associated replicase (Rep), whose amino acid sequence is highly conserved and shows significant homology with various circoviruses [1]. ORF2 encodes the capsid protein (Cap), which is the sole structural protein and exhibits strong immunogenicity [5]. ORF3 is similar in size and location to ORF3 in porcine circovirus type 2 (PCV2) and may encode an apoptotic protein that plays a critical role in viral pathogenesis [6]. Currently, there are three major DuCV genotypes: DuCV-1, DuCV-2, and DuCV-3. DuCV-1 can be further subdivided into subtypes DuCV-1a, DuCV-1b, DuCV-1c, and DuCV-1d, while DuCV-2 can be divided into subtypes DuCV-2a, DuCV-2b, and DuCV-2c [3,7,8]. DuCV-3 is a newly identified genotype discovered in China, in 2022, with limited research conducted on it at present [4].

DuCV was first identified in six-week-old female Mulard ducks in Germany in 2003 [1,9]. It subsequently spread across the world, demonstrating global distribution and high infection rates. For example, infection rates of 84.2% were reported in Hungary [10], 38.2% in Taiwan [11], 10.0–87.2% in China [3,12,13], 43.1% in Vietnam [14], and 54.5% in the United Kingdom [15]. In South Korea, DuCV was first reported by Cha et al. in 2013, with a prevalence of 21.8% during 2011–2012, with DuCV-1b as the dominant genotype [16,17].

DuCV primarily infects domestic ducks, including the Muscovy, Mule, Cherry, Mulard, and Pekin duck. However, it can also infect wild species including the Mallard, Green-winged Teal, and Falcated duck [18,19,20]. Ducks of a wide range of ages are susceptible to DuCV, but those aged 3–5 weeks are particularly vulnerable [10,14]. Horizontal transmission, primarily via the cloacal–fecal–oral route, is considered the primary mode of DuCV transmission; however, it is important to note that vertical transmission through eggs is also possible [2]. Clinically, DuCV infections are often subclinical or manifest with feathering disorders, growth retardation, and immunosuppression, and they are often accompanied by co-infection with other pathogens [9,17,21]. Previous studies have indicated that DuCV infection significantly increases the infection rates of *Riemerella anatipestifer* (RA), *Escherichia coli* (*E. coli*), and duck hepatitis A virus (DHAV) [22]. Additionally, studies have shown that the co-infection of DuCV with novel goose parvovirus-related virus (NGPV) enhances pathogenicity and may lead to clinical symptoms such as duck beak atrophy and dwarfism syndrome (BADS) or feather shedding syndrome (FSS) [23,24]. Furthermore, while using the Korean DuCV-1 strain for experimental infection, Hong et al. demonstrated characteristics of systemic infection, persistent infection, and horizontal transmission [25]. Notable pathological changes were observed in immune organs such as the bursa of Fabricius, thymus, and spleen, while similar findings were reported in a study using the Chinese DuCV-1 strain for experimental infection [26].

The high prevalence and global dissemination of DuCV have caused substantial economic losses in the duck farming industry. Recent studies have shown that DuCV strains from both domestic and wild ducks exhibit high genetic similarity, suggesting that migratory birds may play a crucial role in transmitting the virus [13,19]. Over the past decade, ongoing DuCV mutations and the emergence of new variant strains in China have raised concerns about the potential spread of the virus to South Korea via migratory birds [2]. This risk is particularly significant given South Korea’s rapid and sustained growth in duck production, which has positioned the country among the top ten global producers of duck meat (https://www.fao.org/faostat/en/#rankings/countries_by_commodity; accessed on 20 August 2024). The absence of recent reports regarding the prevalence of DuCV in South Korea renders the potential impact on the current duck industry uncertain [17]. The steady global increase in DuCV prevalence and the complicated infection patterns highlight the need to assess the current status of DuCV infections in South Korea [2]. Therefore, this study aims to conduct an epidemiological investigation of DuCV in samples collected from South Korean farms between 2013 and 2022, in order to understand the prevalence and genetic variation of DuCV in South Korea in recent years.

## 2. Materials and Methods

### 2.1. Sample Collection

**Figure 1:**
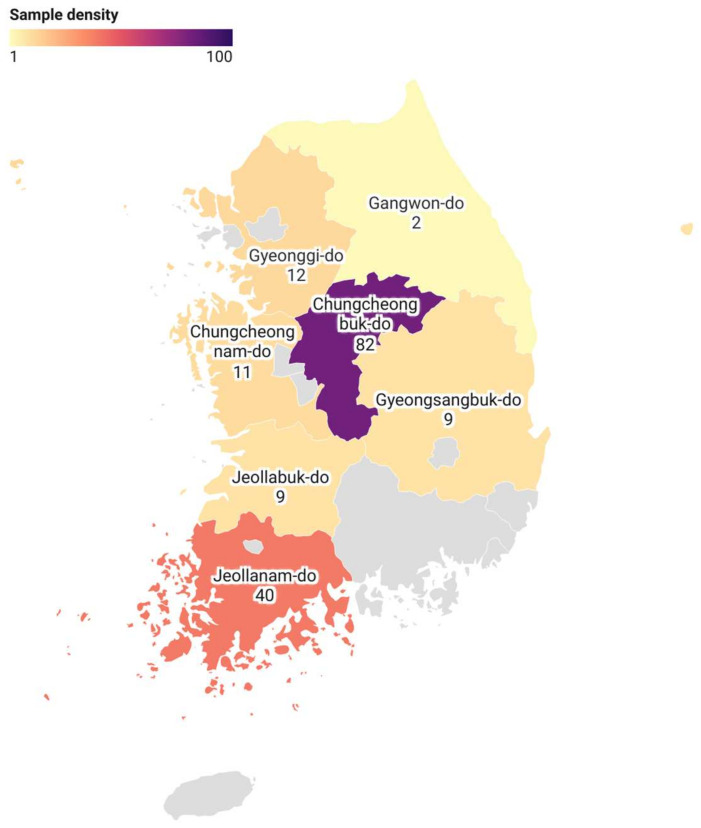
Geographic distribution of DuCV samples tested in South Korea between 2013 and 2022. The number of samples collected varies across different regions, which is reflected by the varying colors. The relationship between sample density and color is indicated by the scale bar.

Samples that were sent to the Center for Avian Disease for disease diagnosis between April 2013 and August 2022 were selected for analysis. The samples were sourced from 184 farms in major duck farming regions of South Korea: 2 farms in Gangwon-do, 12 farms in Gyeonggi-do, 9 farms in Gyeongsangbuk-do, 40 farms in Jeollanam-do, 9 farms in Jeollabuk-do, 11 farms in Chungcheongnam-do, 82 farms in Chungcheongbuk-do, and 19 farms with unspecified information (Figure 1). The sample types included both meat and breeder ducks, with a broad age range (from 1 to 518 days old). Each farm provided between 5 and 20 live or dead ducks, most of which exhibited clinical signs of depression, growth retardation, decreased production performance, or common disease symptoms. After euthanasia by carbon dioxide asphyxiation, necropsies were performed, and samples of the bursa of Fabricius or spleen were collected and preserved at −70 °C for further analysis.

### 2.2. DNA Extraction

Bursa of Fabricius or spleen samples from the same farm were pooled and homogenized to a 20% suspension in sterile PBS containing 1% antibiotic–antimycotic (Invitrogen, Carlsbad, CA, USA). After centrifugation at 13,000 rpm for 10 min at 4 °C, viral DNA was extracted from the supernatant using the Viral Gene-Spin™ Viral DNA/RNA Extraction Kit (iNtRON Biotechnology, Daejeon, Republic of Korea).

### 2.3. Virus Detection and Whole-Genome Amplification

Details of the virus detection and whole-genome amplification primers are provided in Table 1. Samples were tested using previously described DuCV universal detection primers, DuCVaF and DuCVaR, with an expected product size of approximately 408 bp within the Rep gene [21]. The PCR was conducted in a 50 µL reaction volume, including 5 µL of 10X e-Taq Reaction Buffer, 1.25 µL of 2 mM dNTP Mix, 5 µL of each primer (DuCVaF and DuCVaR, 5 pmol), 0.4 µL of Solg™ e-Taq DNA Polymerase (5 U/µL, Solgent, Daejeon, Republic of Korea), 2 µL of DNA, and 31.35 µL of ddH₂O. The PCR cycling conditions were as follows: initial denaturation at 94 °C for 3 min; 35 cycles of 94 °C for 40 s (denaturation), 60 °C for 20 s (annealing), and 72 °C for 2 min (extension); followed by a final extension at 72 °C for 7 min. PCR products were electrophoresed on 1.5% agarose gel. Positive amplification products were purified and sent to Bioneer Corporation (Daejeon, Republic of Korea) for sequencing. The results were further confirmed using NCBI BLAST (https://blast.ncbi.nlm.nih.gov/Blast.cgi; accessed on 23 January 2024).

Whole-genome amplification was performed on a subset of samples that were positive by both PCR and sequencing. The PCR reaction was conducted in a 25 µL reaction mixture comprising 2.5 µL of 10X e-Taq Reaction Buffer, 1.5 µL of 2 mM dNTP Mix, 1 µL each of forward and reverse primers (10 pmol), 0.5 µL of Solg™ e-Taq DNA Polymerase, and 2 µL of DNA, with the remaining volume comprising 16.5 µL of ddH₂O. The PCR conditions were as follows: initial denaturation at 94 °C for 5 min; followed by 35 cycles of denaturation at 94 °C for 30 s, annealing at 53 °C for 1 min, and extension at 72 °C for 1 min; with a final extension at 72 °C for 15 min. Electrophoresis and sequencing were performed using the same methods as described for virus detection. The resulting sequences were processed and assembled using ChromasPro software (ChromasPro 1.7, Technelysium Pty Ltd., Tewantin, Australia).

### 2.4. Sequence Analysis

To understand the genetic characteristics of the currently circulating DuCV in South Korea, a phylogenetic analysis was conducted using 132 DuCV gene sequences uploaded to the NCBI GenBank database from various countries between 2002 and 2023 (Supplementary Table S1) and the DuCV gene sequences isolated in this study. MEGA 7.0 software was utilized to construct phylogenetic trees based on the complete genome using the maximum composite likelihood model and the neighbor-joining (NJ) method with 1000 bootstrap replicates.

MegAlign (DNASTAR Lasergene, version 11) was then used to align the nucleotide and inferred amino acid sequences using the Clustal W method, followed by homology analysis. MEGA 7.0 was then used to statistically analyze and characterize the specific sites in the inferred amino acid sequences of ORF1 and ORF2 from different DuCV genotypes.

To detect potential recombination events among different DuCV strains, seven methods from the RDP v4.101 software (RDP, Chimera, BootScan, 3Seq, GeneConv, MaxChi, SiScan) were used to evaluate the aligned DuCV sequences [28]. Default parameters were employed for the analysis, with recombination considered significant if identified by at least three of the methods. Further confirmation and analyses of the detected recombination events were conducted using SimPlot software (version 3.5.1).

## 3. Results

### 3.1. Epidemiological Investigation

Samples collected from 184 farms in major duck-raising regions across Korea between April 2013 and August 2022 were tested (Table 2). Of these, 54 farms were identified as DuCV-positive, with an overall infection rate of 29.4% (54/184). Positive detections were reported in all regions except Gangwon-do. The highest infection rate was observed in Jeollanam-do, at 37.5% (15/40).

**Figure 2:**
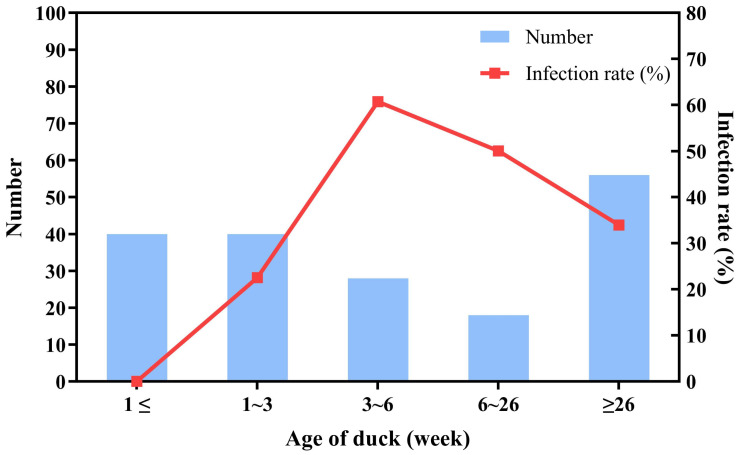
The relationship between the age distribution of DuCV samples and positive infection rates. The blue bars represent the number of samples tested at each age, while the red line represents the positive rate corresponding to each age group.

The 184 tested samples exhibited a wide age range with notable variations in infection rates (Figure 2). Specifically, the infection rate was 0% (0/40) for samples aged ≤1 week, 22.5% (9/40) for samples aged 1–3 weeks, 60.7% (17/28) for samples aged 3–6 weeks, 50% (9/18) for samples aged 6–26 weeks, 33.9% (19/56) for samples aged >26 weeks, and 0% (0/2) for samples of unknown age.

### 3.2. Phylogenetic Analysis

**Figure 3:**
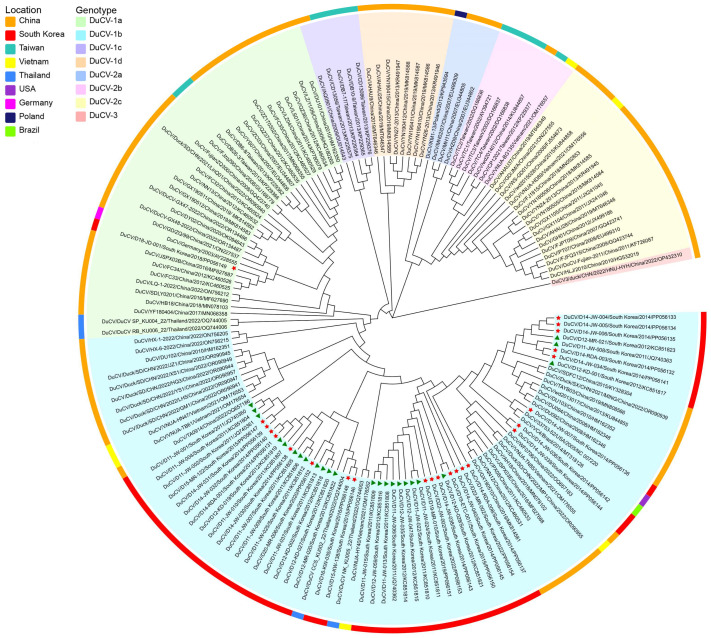
Phylogenetic analysis of complete DuCV genome sequences. The phylogenetic tree was constructed using the neighbor-joining method and the maximum composite likelihood model in MEGA 7.0 (1000 bootstrap replicates), based on the complete genome sequences of Korean DuCV strains and reference sequences submitted to NCBI GenBank from various countries. Different genotypes are denoted by colored backgrounds, and the outer ring indicates the countries from which the strains were isolated, as shown in the legend. Different shape markers preceding the sequences in the tree represent DuCV strains isolated in South Korea during different periods: green triangles indicate 24 Korean DuCV strains previously reported in 2011–2012, and red stars indicate 24 Korean DuCV strains identified in this study from 2013 to 2022.

To analyze the genetic and evolutionary relationships between the circulating DuCV strains in South Korea and other DuCV strains, whole-genome sequencing was performed on 24 out of the 54 positive samples (Supplementary Table S2). A phylogenetic tree based on the whole genome was constructed using the NJ method (Figure 3). The results revealed that the majority of DuCV strains obtained between 2013 and 2022 belong to the DuCV-1b subtype (*n* = 23, 95.8%), which is most closely related to the DuCV strains detected in 2011–2012. DuCV-1b remains the predominant subtype in South Korea.

Additionally, a strain designated as D18-JD-001 (PP056149) was identified as the DuCV-1a genotype, marking the first detection of this subtype in South Korea. This strain was detected in a bursa of Fabricius sample from a 41-day-old meat duck in Chungcheongnam-do in 2018 and showed a close genetic relationship with DuCV-1a strains from China.

### 3.3. Homology Analysis

Homology analysis was conducted on 47 Korean DuCV-1b sequences: 24 were described by Cha et al. (2013) [16] from 2011–2012, and 23 were successfully sequenced in this study. These were compared with sequences from other countries. The nucleotide homology of the whole genome among Korean DuCV-1b strains ranged from 96.0% to 100% (Table 3). The nucleotide and inferred amino acid homology for ORF1 ranged from 97.4% to 100% and 98.3% to 100%, respectively. For ORF2, nucleotide and inferred amino acid homology ranged from 93.8% to 100% and 95.0% to 100%, respectively. Korean DuCV-1b strains showed higher whole-genome nucleotide homology (91.3–99.4%) with DuCV-1b subtype sequences from other countries compared to their homology with DuCV-1a, 1c, and 1d subtype sequences from other countries (92.9–96.9%, 93.3–95.3%, and 91.0–92.2%, respectively). Moreover, they exhibited lower homology with DuCV-2a, 2b, and 2c subtype sequences from other countries (83.7–85.6%, 82.7–84.0%, and 82.3–84.0%, respectively).

Comparison and homology analysis of the Korean DuCV-1a subtype sequences obtained in this study with other sequences in the phylogenetic tree revealed that the Korean DuCV-1a strain is most homologically similar to DuCV-1a strains from other countries. Whole-genome nucleotide homology ranged from 95.8% to 99.6%. For ORF1, nucleotide and inferred amino acid homology ranged from 96.9% to 99.7% and 98.3% to 100%, respectively. For ORF2, nucleotide and inferred amino acid homology ranged from 93.5% to 99.7% and 96.5% to 100%, respectively. Notably, the whole-genome nucleotide homology and ORF2 nucleotide and amino acid homology between Korean DuCV-1a and Korean DuCV-1b were higher compared with other subtypes. Specific data are presented in Table 4.

### 3.4. Amino Acid Mutation Analysis

**Figure 4:**
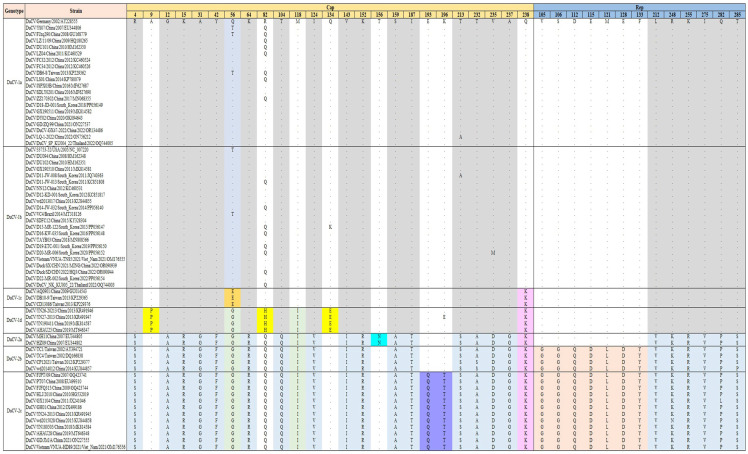
Characteristic amino acid site mutations in the ORF1 (Rep protein) and ORF2 (Cap protein) sequences among different DuCV genotypes or subtypes.

To further understand the characteristics of the inferred ORF1 and ORF2 amino acids in each genotype, one or two sequences isolated from each country or region in different years were selected for amino acid sequence analysis. The results showed specific mutation sites in the amino acid sequences of different genotypes or subtypes (Figure 4). In ORF1, there were six amino acid differences between the DuCV-1 and DuCV-2 genotypes at positions 212 (L to V), 248 (R to K), 255 (K to R), 275 (I to V), 282 (Q to P/L), and 285 (T to S/P). Additionally, the DuCV-2b and DuCV-2c subtypes have unique amino acid mutations distinguishing them from other genotypes at positions 105 (V to G), 106 (S to G), 112 (D to Q), 115 (E to D), 121 (M to L), 128 (E to D), and 133 (F to Y).

By contrast, amino acid mutations in ORF 2 were more frequent and distributed throughout the entire protein. There were 16 amino acid differences between the DuCV-1 and DuCV-2 genotypes, at positions 4 (R to S), 12 (G to A), 15 (K to R), 31 (A to G), 42 (Y to P), 64 (K to R), 104 (T to Q), 124 (I to V), 143 (V to I), 152 (K to R), 159 (S to A), 187 (I to T), 213 (T/A to S), 232 (T to S), 235 (V/M to D), and 237 (A to G). Concurrently, the DuCV-1d subtype exhibited specific characteristic amino acid mutations at positions 9 (A to P), 82 (R/Q to H), and 134 (Q/K to E); the DuCV-2a subtype showed a mutation at position 156 (T to N); and the DuCV-2c subtype had characteristic mutations at positions 193 (E to Q) and 196 (K/E to T).

### 3.5. Recombination Analysis

**Figure 5:**
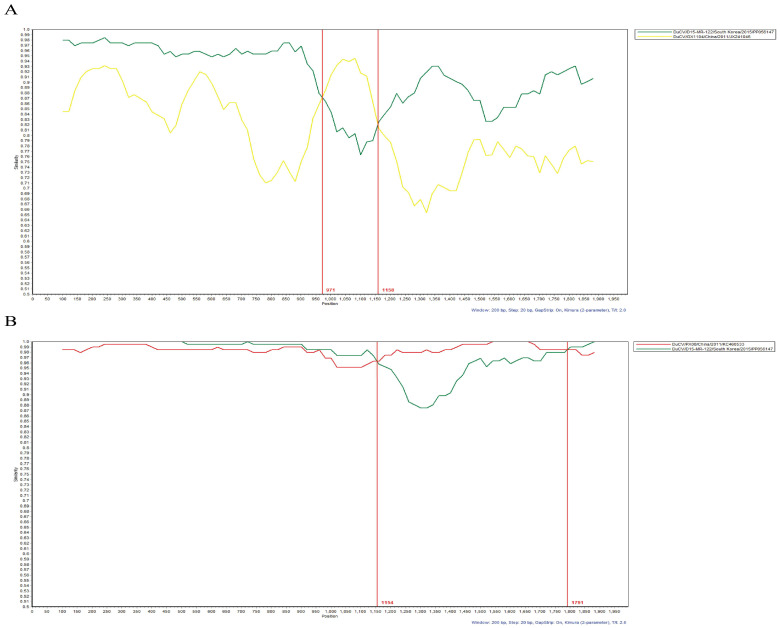
Recombination analysis of representative events involving Korean DuCV-1b subtype isolates based on complete genome sequences. (**A**) Event 2: Korean DuCV-1b subtype D15-MR-122 (PP056147) and Chinese DuCV-2c subtype GX1104 (JX241046) as the putative parental strains of Chinese DuCV-1d subtype AHAU25 (MT646347). (**B**) Event 13: Korean DuCV-1b subtype D15-MR-122 (PP056147) and Chinese DuCV-1b subtype PX08 (KC460533) as the putative parental strains of Korean DuCV-1b subtype D14-JW-036 (PP056142). All analyses were implemented with a window size of 200 bp and a step size of 20 bp using the Kimura 2-parameter model. The X-axis indicates the location of the query sequence, and the Y-axis shows the percentage similarity between the parental sequences and the query sequence.

RDP v4.101 recombination analysis identified 18 recombination events predicted by at least three different methods (Supplementary Table S3). Several Korean DuCV-1b subtype strains were identified as potentially resulting from recombination or playing significant roles in the generation of other recombinant strains. For the recombination events involving the Korean DuCV-1b subtype, specifically Events 2 and 13, SimPlot analysis was employed to further confirm the likelihood of these recombination events occurring. In Event 2, the Korean DuCV-1b strain D15-MR-122 (PP056147) was identified as the major parent, with the Chinese DuCV-2c strain GX1104 (JX241046) as the minor parent, potentially resulting in the formation of the Chinese DuCV-1d strain AHAU25 (MT646347) through recombination (Figure 5A).

In Event 13, the Korean DuCV-1b strain D14-JW-036 (PP056142) is proposed to have originated from recombination between the Korean DuCV-1b strain D15-MR-122 (PP056147) as the major parent and the Chinese DuCV-1b strains GX1104 and JX241046 as the minor parents (Figure 5B). However, there was no evidence of recombination events in the DuCV-1a sequences isolated in this study.

## 4. Discussion

Since DuCV was first reported in Germany in 2003, its infection range has gradually expanded over time, leading to a global distribution with high infection rates [2]. DuCV infection leads to stunted growth in ducks and severe damage to immune organs, increasing the likelihood of secondary infections and exacerbating clinical symptoms of other diseases [23,25,29]. Furthermore, the absence of targeted vaccines and treatments has made controlling DuCV more challenging, posing a significant threat to the duck industry [30,31]. The epidemiological findings indicate an upward trend in DuCV infection rates, increasing from 21.8% in 2011–2012 to 29.4%, coupled with a geographical expansion from western to eastern regions in Korea [17]. Although the duck farming industry in Korea has developed rapidly in recent years, biosecurity measures and hygiene management in duck farms remain relatively inadequate compared to chicken farms [17]. It is also important to note that most duck farms in Korea operate in semi-open environments, which allows ducks to come into frequent contact with wild birds during the rearing process [32]. These factors may contribute to the rising prevalence and expanding geographic distribution of DuCV. Infection rates in South Korea remain below those reported in other countries such as Hungary (84.2%) [10], China (10–87.2%) [3,12,13], Vietnam (43.1%) [14], and the United Kingdom (54.5%) [15]. However, this rising infection trend and geographical diffusion pattern underscores the need for heightened disease surveillance and increased awareness among stakeholders about the potential impacts of DuCV infection in ducks.

This study identified a significant inverse relationship between DuCV infection rates and the age of ducks during the young duck period, revealing a distinct age-dependent pattern. Ducks less than one week old were rarely infected, as the high levels of maternal antibodies provided passive immunity. As the maternal immunoglobulins in duckling serum begin to decrease by 5 days post hatching and reach their lowest levels in around 14 days [33], infection rates gradually increased from 1 to 3 weeks of age. The infection rate peaked at 60.71% in ducks aged 3 to 6 weeks, when maternal antibodies were largely depleted. On the other hand, as the number of infected ducks increased and immune organs matured, ducks began to produce their own antibodies against DuCV, which led to increased resistance and a subsequent decline in infection rates. This pattern of infection rates linked to maternal antibody depletion and immune system maturation is commonly observed in other diseases as well [34,35].

Notably, this research is the first to examine infection rates in breeding ducks older than 26 weeks, revealing a significant infection rate of 33.9%. This finding highlights that ducks of all age groups can be infected with DuCV. The detection of a substantial infection rate in older ducks underscores the potential long-term impact of DuCV, which may affect breeding ducks and compromise reproductive health [25]. Given the possibility of vertical transmission [36], it is essential to investigate the epidemiological relationship between DuCV prevalence in breeder farms and meat duck farms. While these findings are significant, our study focused solely on farm-level infection rates and did not investigate infection at the individual level. Future research should aim to examine infection rates at the individual level within farms to provide a more detailed understanding of DuCV infection. Furthermore, the samples used in this study were collected exclusively from ducks exhibiting clinical symptoms, which may have resulted in an overestimated infection rate. Since DuCV infections are often subclinical, this posed significant challenges during sample collection, as reported in other epidemiological investigations [3,15,20,23,37]. This limitation highlights the need for future research to adopt broader sampling strategies to ensure more comprehensive and representative epidemiological data.

The phylogenetic and homological relationships between the 24 Korean DuCV sequences obtained in this study and the 132 full-genome sequences from the NCBI GenBank database were determined. The full-genome length of the strains analyzed in this study ranged from 1993 to 1996 nt, showing no significant change in nucleotide length compared with the strains measured in 2011–2012 (1993–1995 nt) [16]. The phylogenetic tree results indicate that the predominant genotype of DuCV in South Korea has not changed since 2011–2012 and remains the DuCV-1b subtype. The whole-genome nucleotide sequence homology among Korean DuCV-1b strains ranged from 96% to 100%, indicating genetic stability in Korean DuCV strains.

This study also reports the novel discovery of a Korean DuCV-1a subtype sequence, D18-JD-001 (PP056149), isolated from the bursa of a 41-day-old meat duck in Chungcheongnam-do in 2018. Phylogenetic analysis revealed that the genome sequence shares the closest genetic relationship with the JSPX03B (MF627687) strain, detected in Cherry Valley ducks in Jiangsu Province, China, in 2016, exhibiting a whole-genome nucleotide homology of 99.6% [23]. Given these results, it is reasonable to assume that the Korean DuCV-1a strain was transmitted from China via migratory birds. The potential for wild birds to serve as reservoirs and vectors for DuCV transmission has been increasingly recognized in recent years, with evidence that wild birds can carry genetically similar DuCV strains to those found in domestic ducks [19]. Wild birds, particularly migratory birds, can travel long distances, thereby potentially introducing viruses to new areas [19]. Also, migratory birds could play a crucial role in the transmission of viral diseases among these countries, such as influenza A virus [38]. It is also with the evidence that the Jiangsu province is located in the eastern coastal area in China, which is part of the East Asian–Australian migratory bird flyway with Korea [2]. However, despite the potential role of wild birds in DuCV transmission, research on DuCV prevalence and genetic diversity in wild bird populations remains significantly limited compared with studies on domestic ducks. This knowledge gap highlights a critical area for future research. Furthermore, to mitigate the potential risks of DuCV-1a to the duck farming industry in Korea, priority must be given to studying its pathogenicity. A comprehensive geographical survey of the current distribution and prevalence of this subtype within Korea is essential, with a particular focus on areas with high duck farming density and known migratory bird stopover sites. Proactive measures will be critical in preventing potential outbreaks and safeguarding the poultry industry.

This study conducted a comprehensive analysis of the amino acid variation characteristics across all DuCV sequences from multiple countries between 2002 and 2022. Previous reports indicated that the primary variations in ORF1 occur in two regions (amino acids 93–133 and 274–285), while amino acid variations in ORF2 are more frequent and distributed throughout the protein, with six major variable regions (amino acids 3–15, 31–63, 104–124, 143–159, 177–213, and 232–238). These findings are closely aligned with our statistical results [7,12]. However, unlike previous studies, our study provides a more detailed analysis of the specific amino acid sites for each genetic subtype. No comprehensive research has thus far been undertaken on the amino acid changes in DuCV that lead to changes in protein function, so the significance of these specific amino acid sites for each genotype remains unclear. Nevertheless, it may be possible to use our findings to develop diagnostic methods that can distinguish between different gene subtypes by targeting single nucleotide polymorphisms (SNPs) that cause amino acid changes in the future, thereby enabling more efficient clinical diagnoses of DuCV [39].

In this study, the ORF2 amino acid sequences of the 23 DuCV-1b strains we obtained were compared to the Korean DuCV-1b strain D11-JW-008 (JQ740363), which was previously used in pathogenicity studies. Several amino acid variations were found to be located within predicted linear B-cell epitopes [2] (Supplementary Table S4). The mutations were located within predicted epitopes A, B, C, E, and F, with three additional mutations found outside the B-cell linear epitopes. Notably, all sequences analyzed in this study shared a common mutation at position 190, where glycine (G) replaced arginine (R), although the cause and implications of this mutation remain unclear. It is hypothesized that differences in key epitopes between recent and historical DuCV strains may reflect viral evolution. Similar to previous findings in PCV, changes in amino acid residues within B-cell linear epitopes of the capsid protein may contribute to antibody-mediated immune escape and alterations in immunogenicity [40]. Viral evolution often leads to increased fitness through beneficial mutations driven by environmental selection pressures [41]. Although no vaccines have been developed for DuCV, the observed shifts in the antigenic profile of B-cell linear epitopes compared with earlier wild-type strains could suggest some level of immune pressure exerted by natural infections with wild-type DuCV [2]. Given the current limited levels of research on DuCV, future studies should focus on the role of these amino acid substitutions in B-cell linear epitopes and their effects on the immune response.

Genetic recombination plays a crucial role in viral evolution, contributing significantly to the genetic diversity of DuCV and leading to the emergence of new isolates or genetic subtypes [8,42]. Our analysis of detected recombination events provides evidence of extensive genetic exchanges occurring during the evolution of DuCV. Notably, these recombination events are not confined within national boundaries but occur across different countries and even continents. Of the 18 recombination events identified in our study (Supplementary Table S3), eight involved DuCV strains from different countries, highlighting the global nature of these genetic exchanges. A particularly intriguing example is the recombination event (Event 1) that likely gave rise to the DuCV-2a strain KM1-13 (KP943594), detected in marine ducks in Poland in 2013. Our analysis suggests this strain resulted from recombination between the American DuCV-1b strain 33753-52 (NC_007220.1) as the primary parent and the Chinese DuCV-2c strain AHAU37 (MT646349) as the minor parent. This intercontinental recombination event underscores the potential for long-distance genetic exchange in DuCV. The observed pattern of recombination suggests that wild birds, particularly migratory species, play a multifaceted role in DuCV epidemiology, findings which align with previous studies reporting the presence and recombination events of DuCV in migrating wild ducks [19]. Our results may suggest that not only do migrating wild birds serve as carriers of DuCV, facilitating its worldwide transmission, but they also contribute significantly to gene flow and recombination with local strains. Furthermore, our results also suggest a complex interplay between DuCV viral strains circulating in domestic duck populations and those carried by wild birds. With the occurrence of such diverse recombination events, both within and across geographical boundaries, future research should focus on elucidating the mechanisms and frequency of these recombination events.

## 5. Conclusions

In summary, this study provides a comprehensive epidemiological survey of DuCV in Korea from 2013 to 2022, including whole-genome sequencing and genetic evolutionary analysis of the obtained sequences. The epidemiological results indicate a gradual increase in both the infection rate and geographical distribution of DuCV across Korea. Notably, infections persist throughout all the growth stages in ducklings and continue to affect breeding ducks, suggesting a complex transmission pattern within duck populations. Genetic evolutionary analysis revealed that DuCV-1b remains the predominant subtype in Korea and identified the emergence of DuCV-1a, which has a high genetic similarity to a Chinese isolate. This suggests the potential introduction of a viral strain from migratory birds, highlighting the role of wild avian populations in virus transmission. Given that intercontinental recombination events underscore the potential for long-distance genetic exchange in DuCV via migratory birds, there is a pressing need to establish a comprehensive cross-regional monitoring program to evaluate their role in virus transmission and mitigate the threat of exotic genotypes to local duck populations. Future research should prioritize intensified surveillance of DuCV, particularly focusing on its evolution driven by recombination, as this process could accelerate viral mutation and potentially affect its virulence. Additionally, developing in vitro culture systems for DuCV will play a crucial role in facilitating deeper exploration of its pathogenic mechanisms. As no effective treatments or vaccines for DuCV are currently available, reducing viral transmission relies solely on enhancing hygiene management and enforcing strict biosecurity measures in duck farms. In conclusion, this comprehensive study significantly advances our understanding of DuCV epidemiology in Korea and provides valuable insights for developing related control strategies.

## Figures and Tables

**Table 1:** Primers for duck circovirus detection and whole-genome sequencing.

Primer	Sequences (5′–3′)	Location *	Size (bp)	Reference
DuCVaF	MGAGCTGCCGCCCTTGAG	238–255	408	[21]
DuCVaR	TCCCGAGTAACCGTCCCACCAC	624–645		
DuCV-FLA1-F	ACTGCAATGGCGAAGAG	43–59	418	In this study
DuCV-FLA1-R	CATAAGTCGTGGGGAACT	443–460		
DuCV-P1-F	TTGAAGAGTCGCTGGGAGGAA	251–271	518	[27]
DuCV-P1-R	CTTAGCAACAAACTGGGTCA	749–768		
DuCV-FLA2-F	ATGCATTTGAATTTCCCGCC	575–594	818	In this study
DuCV-FLA2-R	GTACTTCGTACCTAAGCC	1375–1392		
DuCV-FLA3-F	CTCATGCCCATGCCGTAATG	1258–1277	724	In this study
DuCV-FLA3-R	CGCTTGTGCGGTCTTTTAT	1963–1981		
DuCV-P3-F	GTAGCCTTCGTCTTCTGAGT	1847–1866	468	[27]
DuCV-P3-R	TATTCTTCATTATCTTCGTCA	300–320		

* The location was determined according to D12-MR-020 (KC851822.1).

**Table 2:** Results of the DuCV epidemiological survey in Korea from 2013 to 2022.

	Region								
	GW *	GG *	GB *	JN *	JB *	CN *	CB *	Unknown	Total
Detection samples	2	12	9	40	9	11	82	19	184
Infection samples	0	4	1	15	3	3	24	4	54
Infection rate	0%	33.3%	11.1%	37.5%	30.0%	27.3%	29.3%	21.1%	29.4%

* GW (Gangwon-do), GG (Gyeonggi-do), GB (Gyeongsangbuk-do), JN (Jeollanam-do), JB (Jeollabuk-do), CN (Chungcheongnam-do), CB (Chungcheongbuk-do).

**Table 3:** Homology analysis between sequences of Korean genotype 1b and other genotypes.

	Complete Genome	ORF1		ORF2	
	nt	nt	aa	nt	aa
Genotype 1b (South Korea)	96.0~100%	97.4~100%	98.3~100%	93.8~100%	95.0~100%
Genotype 1b (other countries)	91.3~99.4%	96.3~99.5%	97.3~100%	89.6~99.4%	91.1~100%
Genotype 1a	92.9~96.9%	95.9~99.3%	97.6~100%	88.2~94.1%	94.2~99.2%
Genotype 1c	93.3~95.3%	96.7~98.8%	98.3~100%	87.7~90.3%	93.4~97.3%
Genotype 1d	91.0~92.2%	95.9~97.3%	98.3~99.7%	88.1~90.2%	92.6~95.0%
Genotype 2a	83.7~85.6%	88.4~92.6%	92.2~97.6%	78.7~82.6%	87.2~92.2%
Genotype 2b	82.7~84.0%	87.3~88.3%	92.8~95.6%	78.2~81.3%	85.7~89.5%
Genotype 2c	82.3~84.0%	87.0~89.8%	92.2~95.6%	77.9~80.1%	84.9~88.4%

**Table 4:** Homology analysis of Korean genotype 1a and other genotypes.

	Complete Genome	ORF1		ORF2	
	nt	nt	aa	nt	aa
Genotype 1a	95.8~99.6%	96.9~99.7%	98.3~100%	93.5~99.7%	96.5~100%
Genotype 1b (South Korea)	94.1~95.4%	96.8~97.6%	99.0~100%	90.6~92.6%	95.0~97.7%
Genotype 1b (other countries)	90.7~95.2%	95.9~98.1%	98.3~100%	86.4~92.9%	91.5~98.1%
Genotype 1c	94.4~95.0%	97.1~98.1%	99.3~100%	90.6~91.6%	95.0~96.9%
Genotype 1d	92.6~93.5%	96.5~97.1%	99.3~99.7%	92.0~92.5%	94.2~95.0%
Genotype 2a	84.2~85.1%	88.3~92.9%	93.2~97.6%	78.7~82.9%	88.4~91.1%
Genotype 2b	82.2~82.7%	87.3~87.9%	93.9~94.9%	77.6~78.9%	87.6~88.8%
Genotype 2c	82.0~82.8%	87.0~88.9%	93.2~94.9%	77.9~78.7%	86.4~88.0%

## Data Availability

The data presented in this study are available from the corresponding authors on reasonable request.

## Supplementary Materials

The following supporting information can be downloaded at: https://www.mdpi.com/article/10.3390/ani14243630/s1, Supplementary Table S1. Reference sequence information used in phylogenetic analysis of DuCV. Supplementary Table S2. Sequence information of 24 DuCV strains obtained in this study. Supplementary Table S3. Predicted recombination events in this study. Supplementary Table S4. Amino acid variations in ORF2 between 23 DuCV-1b strains and D11-JW-008 within predicted B-cell epitopes.
