# Supplementary material for: Epidemiological Investigation and Genetic Analysis of Duck Circovirus in Korea from 2013 to 2022
Source: Animals (Basel). 2024 Dec 16;14(24):3630. doi: 10.3390/ani14243630 (PMC11672762; doi:10.3390/ani14243630)
Supplement: Supplementary file 1 [file animals-14-03630-s001.zip › animals-3283540-supplementary.pdf]

Table S1. Scheme 1. Reference sequence information used in phylogenetic analysis of DuCV.

NO.	GenBank NO.	Strains name	Names in phylogenetic tree	Host	Genome size(nt)
1	JQ740360.1	D11-JW-001	DuCV/D11-JW-001/South Korea/2011/JQ740360	Duck	1993
2	JQ740361.1	D11-JW-002	DuCV/D11-JW-002/South Korea/2011/JQ740361	Duck	1993
3	KC851804.1	D11-JW-004	DuCV/D11-JW-004/South Korea/2011/KC851804	Duck	1993
4	JQ740362.1	D11-JW-006	DuCV/D11-JW-006/South Korea/2011/JQ740362	Duck	1995
5	KC851805.1	D11-JW-007	DuCV/D11-JW-007/South Korea/2011/KC851805	Duck	1994
6	JQ740363.1	D11-JW-008	DuCV/D11-JW-008/South Korea/2011/JQ740363	Duck	1993
7	KC851806.1	D11-JW-009	DuCV/D11-JW-009/South Korea/2011/KC851806	Duck	1994
8	KC851807.1	D11-JW-010	DuCV/D11-JW-010/South Korea/2011/KC851807	Duck	1994
9	KC851808.1	D11-JW-013	DuCV/D11-JW-013/South Korea/2011/KC851808	Duck	1995
10	KC851809.1	D11-JW-015	DuCV/D11-JW-015/South Korea/2011/KC851809	Duck	1995
11	KC851810.1	D11-JW-022	DuCV/D11-JW-022/South Korea/2011/KC851810	Duck	1995
12	KC851811.1	D11-JW-024	DuCV/D11-JW-024/South Korea/2011/KC851811	Duck	1995
13	KC851812.1	D11-JW-025	DuCV/D11-JW-025/South Korea/2011/KC851812	Duck	1994
14	KC851813.1	D11-JW-037	DuCV/D11-JW-037/South Korea/2011/KC851813	Duck	1993
15	KC851814.1	D12-JW-035	DuCV/D12-JW-035/South Korea/2012/KC851814	Duck	1995
16	KC851815.1	D12-JW-047	DuCV/D12-JW-047/South Korea/2012/KC851815	Duck	1995
17	KC851816.1	D12-JW-059	DuCV/D12-JW-059/South Korea/2012/KC851816	Duck	1995
18	KC851817.1	D12-KD-001	DuCV/D12-KD-001/South Korea/2012/KC851817	Duck	1994
19	KC851818.1	D12-KD-002	DuCV/D12-KD-002/South Korea/2012/KC851818	Duck	1994
20	KC851819.1	D12-KD-019	DuCV/D12-KD-019/South Korea/2012/KC851819	Duck	1994
21	KC851820.1	D12-KD-027	DuCV/D12-KD-027/South Korea/2012/KC851820	Duck	1994
22	KC851821.1	D12-KD-028	DuCV/D12-KD-028/South Korea/2012/KC851821	Duck	1995
23	KC851822.1	D12-MR-020	DuCV/D12-MR-020/South Korea/2012/KC851822	Duck	1994

24	KC851823.1	D12-MR-021	DuCV/D12-MR-021/South Korea/2012/KC851823	Duck	1994
25	EU344806.1	YS07	DuCV/YS07/China/2007/EU344806	Cherry valley duck	1992
26	EU344807.1	ZC03	DuCV/ZC03/China/2007/EU344807	Muscovy duck	1992
27	EU499309.1	MH02/07	DuCV/MH02/07/China/2007/EU499309	M18 Mule duck	1988
28	EU344805.1	MH11	DuCV/MH11/China/2007/EU344805	Muscovy duck	1988
29	EU344802.1	HZ09	DuCV/HZ09/China/2007/EU344802	Muscovy duck	1989
30	GQ423741.1	FJPT09	DuCV/FJPT09/China/2007/GQ423741	Muscovy duck	1988
31	HM162348.1	DU094	DuCV/DU094/China/2008/HM162348	Cherry valley pekin duck	1993
32	HM162346.1	DU092	DuCV/DU092/China/2008/HM162346	Cherry valley pekin duck	1993
33	GQ423740.1	FujianZQ300	DuCV/FujianZQ300/China/2008/GQ423740	Muscovy duck	1996
34	GU168779.1	FJzq290	DuCV/FJzq290/China/2008/GU168779	Muscovy duck, Grimaud	1995
35	FJ554673.1	WS-GD01	DuCV/WS-GD01/China/2008/FJ554673	Muscovy duck	1988
36	EU499310.1	PT07	DuCV/PT07/China/2008/EU499310	Muscovy duck	1988
37	HQ180265.1	LZ/11/09	DuCV/LZ/11/09/China/2009/HQ180265	Muscovy duck	1994
38	GU014543.1	AQ0901	DuCV/AQ0901/China/2009/GU014543	Duck	1995
39	GQ423744.1	FJFQ315	DuCV/FJFQ315/China/2009/GQ423744	Muscovy duck	1988
40	HM162352.1	DU103	DuCV/DU103/China/2010/HM162352	Cherry valley pekin duck	1993
41	HM162351.1	DU102	DuCV/DU102/China/2010/HM162351	Cherry valley pekin duck	1996
42	HM162350.1	DU101	DuCV/DU101/China/2010/HM162350	Cherry valley pekin duck	1994
43	HG532019.1	HLJ/2010	DuCV/HLJ/2010/China/2010/HG532019	Duck	1988
44	KC460533.1	PX08/2011	DuCV/PX08/China/2011/KC460533	Duck	1994
45	KC460529.1	LZ04/2011	DuCV/LZ04/China/2011/KC460529	Duck	1995
46	JX241045.1	GX1105	DuCV/GX1105/China/2011/JX241045	Duck	1988
47	JX241046.1	GX1104	DuCV/GX1104/China/2011/JX241046	Duck	1988
48	KF726087.1	DuCV-Fujian-2011	DuCV/DuCV-Fujian-2011/China/2011/KF726087	Anas platyrhynchos	1988
49	KC460531.1	NN12/2012	DuCV/NN12/China/2012/KC460531	Duck	1993

50	KC460525.1	FC33/2012	DuCV/FC33/China/2012/KC460525	Duck	1993
51	KC460526.1	FC34/2012	DuCV/FC34/China/2012/KC460526	Duck	1993
52	KC460532.1	NN13/2012	DuCV/NN13/China/2012/KC460532	Duck	1995
53	KC460524.1	FC32/2012	DuCV/FC32/China/2012/KC460524	Duck	1995
54	KC460535.1	QZ37/2012	DuCV/QZ37/China/2012/KC460535	Duck	1994
55	KC460527.1	FC35/2012	DuCV/FC35/China/2012/KC460527	Duck	1994
56	KC460534.1	QZ36/2012	DuCV/QZ36/China/2012/KC460534	Duck	1995
57	JX499186.1	GH01	DuCV/GH01/China/2012/JX499186	Muscovy duck	1988
58	KU844855.1	wd2013017	DuCV/wd2013017/China/2013/KU844855	Anas platyrhynchos	1993
59	KR491947.1	YN27-2013	DuCV/YN27-2013/China/2013/KR491947	Muscovy duck	1987
60	KR491946.1	YN26-2013	DuCV/YN26-2013/China/2013/KR491946	Muscovy duck	1987
61	KR491945.1	YN24-2013	DuCV/YN24-2013/China/2013/KR491945	Muscovy duck	1988
62	KP780079.1	LS01	DuCV/LS01/China/2014/KP780079	Muscovy duck	1993
63	KU844857.1	wd2014012	DuCV/wd2014012/China/2014/KU844857	Anas crecca	1988
64	KY328304.1	SDFC12	DuCV/SDFC12/China/2015/KY328304	Cherry Valley Pekin duck	1993
65	KU844858.1	wd2015028	DuCV/wd2015028/China/2015/KU844858	Mareca falcata	1988
66	MF627688.1	JSPX03E	DuCV/JSPX03E/China/2016/MF627688	Cherry Valley duck	1993
67	MF627690.1	SDLY0201	DuCV/SDLY0201/China/2016/MF627690	Cherry Valley duck	1995
68	MF627687.1	JSPX03B	DuCV/JSPX03B/China/2016/MF627687	Cherry Valley duck	1993
69	MN068358.1	YF180404	DuCV/YF180404/China/2017/MN068358	Cherry valley duck	1995
70	MN068355.1	ZZ170302	DuCV/ZZ170302/China/2017/MN068355	Cherry valley duck	1994
71	MN078102.1	AH18	DuCV/AH18/China/2018/MN078102	Duck	1994
72	MN078103.1	HB18	DuCV/HB18/China/2018/MN078103	Duck	1992
73	MN052853.1	FJ1815	DuCV/FJ1815/China/2018/MN052853	Muscovy duck	1988
74	MK814585.1	YN180506	DuCV/YN180506/China/2018/MK814585	Cherry Valley Peking duck	1988

75	MK814584.1	YN180505	DuCV/YN180505/China/2018/MK814584	Muscovy duck	1988
76	MN808566.1	TAYB03	DuCV/TAYB03/China/2019/MN808566	Cherry valley duck	1995
77	MK814581.1	GX190510	DuCV/GX190510/China/2019/MK814581	Cherry Valley Peking duck	1993
78	MK814583.1	GX190512	DuCV/GX190512/China/2019/MK814583	Muscovy duck	1995
79	MK814582.1	GX190511	DuCV/GX190511/China/2019/ MK814582	Cherry Valley Peking duck	1995
80	MT646346.1	AHAU9	DuCV/AHAU9/China/2019/MT646346	Duck	1988
81	MT646347.1	AHAU25	DuCV/AHAU25/China/2019/MT646347	Duck	1987
82	MK814589.1	YN190415	DuCV/YN190415/China/2019/MK814589	Mallard	1987
83	MK814588.1	YN190412	DuCV/YN190412/China/2019/MK814588	Muscovy duck	1987
84	MK814587.1	YN190411	DuCV/YN190411/China/2019/MK814587	Mulard duck	1987
85	MK814586.1	YN190410	DuCV/YN190410/China/2019/MK814586	Muscovy duck	1987
86	MT646349.1	AHAU37	DuCV/AHAU37/China/2019/MT646349	Duck	1991
87	MT646348.1	AHAU28	DuCV/AHAU28/China/2019/MT646348	Duck	1988
88	OK094643.1	DY02	DuCV/DY02/China/2020/OK094643	Duck	1994
89	ON227537.1	GD/ZQ/99	DuCV/GD/ZQ/99/China/2021/ON227537	Duck	1996
90	ON227555.1	GD/JM/A	DuCV/GD/JM/A/China/2021/ON227555	Duck	1988
91	OR090939.1	DuCV/Duck/SX/CHN/2021/MING	DuCV/Duck/SX/CHN/2021/MING/China/2022/OR090939	Duck	1994
92	OQ657183.1	WF0706	DuCV/WF0706/China/2022/OQ657183	Cherry valley duck	1993
93	OR090955.1	DuCV/Duck/SD/CHN/2022/MP1	DuCV/Duck/SD/CHN/2022/MP1/China/2022/OR090955	Duck	1990
94	OQ657186.1	TA0914	DuCV/TA0914/China/2022/OQ657186	Cherry valley duck	1993
95	OR090941.1	DuCV/Duck/SD/CHN/2022/GM1	DuCV/Duck/SD/CHN/2022/GM1/China/2022/OR090941	Duck	1900
96	OR090947.1	DuCV/Duck/SD/CHN/2022/LH5	DuCV/Duck/SD/CHN/2022/LH5/China/2022/OR090947	Duck	1990
97	OR090957.1	DuCV/Duck/SD/CHN/2022/YS1	DuCV/Duck/SD/CHN/2022/YS1/China/2022/OR090957	Duck	1989
98	OR090944.1	DuCV/Duck/SD/CHN/2022/HQ3	DuCV/Duck/SD/CHN/2022/HQ3/China/2022/OR090944	Duck	1989
99	OR090949.1	DuCV/Duck/SD/CHN/2022/XS1	DuCV/Duck/SD/CHN/2022/XS1/China/2022/OR090949	Duck	1989

100	OR090945.1	DuCV/Duck/SD/CHN/2022/JZ1	DuCV/Duck/SD/CHN/2022/JZ1/China/2022/OR090945	Duck	1991
101	ON756215.1	HX-6-2022	DuCV/HX-6-2022/China/2022/ON756215	Duck	1993
102	ON756205.1	HX-1-2022	DuCV/HX-1-2022/China/2022/ON756205	Duck	1993
103	ON756212.1	LQ-1-2022	DuCV/LQ-1-2022/China/2022/ON756212	Duck	1995
104	OR134487.1	DuCV-GX48-2022	DuCV/DuCV-GX48-2022/China/2022/OR134487	Duck	1994
105	OR134486.1	DuCV-GX47-2022	DuCV/DuCV-GX47-2022/China/2022/OR134486	Duck	1994
106	OR090940.1	DuCV/Duck/SD/CHN/2021/LinQ01	DuCV/Duck/SD/CHN/2021/LinQ01/China/2022/OR090940	Duck	1995
107	OP432310.1	DuCV3/duck/CHN/2022/HNU-HYH	DuCV3/duck/CHN/2022/HNU-HYH/China/2022/OP432310	Duck	1755
108	AY394721.1	TC1/2002	DuCV/TC1/Taiwan/2002/AY394721	Duck	1988
109	DQ166836.1	TC2/2002	DuCV/TC2/Taiwan/2002/DQ166836	Duck	1988
110	DQ166837.1	TC3/2002	DuCV/TC3/Taiwan/2002/DQ166837	Duck	1988
111	DQ166838.1	TC4/2002	DuCV/TC4/Taiwan/2002/DQ166838	Duck	1988
112	KP229377.1	CP12021	DuCV/CP12021/Taiwan/2012/KP229377	Muscovy duck	1988
113	KP229366.1	DB26-3	DuCV/DB26-3/Taiwan/2013/KP229366	Mule duck	1993
114	KP229362.1	DB6-8	DuCV/DB6-8/Taiwan/2013/KP229362	Pekin duck	1992
115	KP229375.1	CD13056	DuCV/CD13056/Taiwan/2013/KP229375	Pekin duck	1995
116	KP229364.1	DB7-17	DuCV/DB7-17/Taiwan/2013/KP229364	Mule duck	1995
117	KP229365.1	DB10-9	DuCV/DB10-9/Taiwan/2013/KP229365	Mule duck	1995
118	KP229376.1	CD13086	DuCV/CD13086/Taiwan/2013/KP229376	Mule duck	1995
119	OQ744003.1	DuCV_NK_KU005_22	DuCV/DuCV NK KU005 22/Thailand/2022/OQ744003	Duck	1996
120	OQ744004.1	DuCV_CCS_KU002_22	DuCV/DuCV CCS KU002 22/Thailand/2022/OQ744004	Duck	1995
121	OQ744006.1	DuCV_RB_KU006_22	DuCV/DuCV RB KU006 22/Thailand/2022/OQ744006	Duck	1995
122	OQ744005.1	DuCV_SP_KU004_22	DuCV/DuCV SP KU004 22/Thailand/2022/OQ744005	Duck	1995
123	OM176555.1	Vietnam/VNUA-TN85/2021	DuCV/VNUA-TN85/Vietnam/2021/OM176555	Duck	1994
124	OM176552.1	Vietnam/VNUA-HY40/2021	DuCV/NUA-HY40/Vietnam/2021/OM176552	Duck	1995

125	OM176554.1	Vietnam/VNUA-TB61/2021	DuCV/VNUA-TB61/Vietnam/2021/OM176554	Duck	1993
126	OM176553.1	Vietnam/VNUA-HN47/2021	DuCV/VNUA-HN47/Vietnam/2021/OM176553	Duck	1993
127	OM176557.1	Vietnam/VNUA-BG135/2021	DuCV/VNUA-BG135/Vietnam/2021/OM176557	Duck	1988
128	OM176556.1	Vietnam/VNUA-HD89/2021	DuCV/VNUA-HD89/Vietnam/2021/OM176556	Duck	1988
129	AY228555.1	/	DuCV/Germany/2003/AY228555	Duck	1996
130	NC_007220.1	33753-52	DuCV/33753-52/USA/2005/NC_007220	Pekin duck	1991
131	KP943594.1	KM1-13	DuCV/KM1-13/Poland/2013/KP943594	Melanitta fusca	1988
132	MT318126.1	VC4	DuCV/VC4/Brazil/2014/MT318126	Anas platyrhynchos	1993

Table S2. Sequence information of 24 DuCV strains obtained in this study

NO.	GenBank NO.	Strain	Collection date	Genome size(nt)
1	PP056131	DuCV/D14-RDA-001/South Korea/2014	10-Oct-2014	1994
2	PP056132	DuCV/D14-RDA-003/South Korea/2014	13-Oct-2014	1993
3	PP056133	DuCV/D14-JW-004/South Korea/2014	24-Oct-2014	1994
4	PP056134	DuCV/D14-JW-005/South Korea/2014	24-Oct-2014	1994
5	PP056135	DuCV/D14-JW-006/South Korea/2014	24-Oct-2014	1994
6	PP056136	DuCV/D14-JW-007/South Korea/2014	24-Oct-2014	1994
7	PP056137	DuCV/D14-RDA-008/South Korea/2014	3-Nov-2014	1994
8	PP056138	DuCV/D14-JW-030/South Korea/2014	20-Nov-2014	1994
9	PP056139	DuCV/D14-JW-031/South Korea/2014	20-Nov-2014	1993
10	PP056140	DuCV/D14-JW-032/South Korea/2014	20-Nov-2014	1994
11	PP056141	DuCV/D14-JW-034/South Korea/2014	20-Nov-2014	1994
12	PP056142	DuCV/D14-JW-036/South Korea/2014	20-Nov-2014	1993
13	PP056143	DuCV/D14-JW-038/South Korea/2014	21-Nov-2014	1995
14	PP056144	DuCV/D14-JW-039/South Korea/2014	21-Nov-2014	1994
15	PP056145	DuCV/D14-JW-040/South Korea/2014	21-Nov-2014	1993
17	PP056146	DuCV/D15-KW-138/South Korea/2015	12-Aug-2015	1994
16	PP056147	DuCV/D15-MR-122/South Korea/2015	6-Jul-2015	1993
18	PP056148	DuCV/D16-KW-035/South Korea/2016	13-Jul-2016	1994
19	PP056149	DuCV/D18-JD-001/South Korea/2018	16-Aug-2018	1993
20	PP056150	DuCV/D19-ETC-001//South Korea/2019	22-Apr-2019	1995
21	PP056151	DuCV/D19-MR-010/South Korea/2019	28-May-2019	1995
22	PP056152	DuCV/D20-MR-006/South Korea/2020	6-Jul-2020	1995
23	PP056153	DuCV/D22-JW-002/South Korea/2022	14-Jul-2022	1996
24	PP056154	DuCV/D22-MR-002/South Korea/2022	1-Jun-2022	1995

Table S3. Predicted recombination events in this study

Event number	Found in	Recombinant strain	Major parent	Minor parent	Detection methods						
					R	G	B	M	C	S	T
1	1	DuCV/KM1-13/Poland/2013/KP943594	DuCV/33753-52/USA/2005/NC_007220	DuCV/AHAU37/China/2019/MT646349	+	+	-	+	+	-	-
2	8	DuCV/AHAU25/China/2019/MT646347	DuCV/D15-MR-122/South Korea/2015/PP056147	DuCV/GX1104/China/2011/JX241046	+	+	+	+	+	-	+
3	1	DuCV/AHAU9/China/2019/MT646346	DuCV/AHAU25/China/2019/MT646347	DuCV/GX1104/China/2011/JX241046	+	+	+	+	+	-	+
4	1	DuCV/HB18/China/2018/MN078103	DuCV/D14-JW-036/South Korea/2014/PP056142	Unknow	+	-	-	+	+	+	+
5	3	DuCV/HZ09/China/2007/EU344802	DuCV/FC32/China/2012/KC460524	DuCV/GX1104/China/2011/JX241046	+	+	+	+	-	+	+
6	10	DuCV/FujianZQ300/China/2008/GQ423740	DuCV/FJzq290/China/2008/GU168779	Unknow	-	-	-	+	-	+	+
7	18	DuCV/GD/ZQ/99/China/2021/ON227537	Unknow	DuCV/WF0706/China/2022/OQ657183	+	+	+	+	-	+	+
8	13	DuCV/D14-JW-040/South Korea/2014/PP056145	DuCV/GX190510/China/2019/MK814581	Unknow	-	-	-	+	-	+	+
9	1	DuCV/AQ0901/China/2009/GU014543	DuCV/SDLY0201/China/2016/MF627690	DuCV/DU094/China/2008/HM162348	+	-	-	+	+	+	+
10	2	DuCV/D22-JW-002/South Korea/2022/PP056153	DuCV/D11-JW-002/South Korea/2011/JQ740361	Unknow	+	+	-	-	-	+	+
11	1	DuCV/AH18/China/2018/MN078102	DuCV/TC1/Taiwan/2002/AY394721	DuCV/JSPX03E/China/2016/MF627688	+	+	-	+	+	+	+
12	1	DuCV/AH18/China/2018/MN078102	DuCV/PX08/China/2011/KC460533	Unknow	+	-	+	+	+	+	-
13	12	DuCV/D14-JW-036/South Korea/2014/PP056142	DuCV/D15-MR-122/South Korea/2015/PP056147	DuCV/PX08/China/2011/KC460533	-	-	-	+	+	+	+
14	37	DuCV/HX-6-2022/China/2022/ON756215	DuCV/D14-JW-036/South Korea/2014/PP056142	DuCV/LQ-1-2022/China/2022/ON756212	-	-	-	+	+	+	+
15	1	DuCV/D22-JW-002/South Korea/2022/PP056153	DuCV/D22-MR-002/South Korea/2022/PP056154	Unknow	-	-	-	+	-	+	+
16	2	DuCV/DuCV RB KU006 22/Thailand/2022/OQ744006	DuCV/GX190510/China/2019/MK814581	DuCV/SDLY0201/China/2016/MF627690	-	-	-	+	-	+	+

17	1	DuCV/DB26-3/Taiwan/2013/KP229366	DuCV/CD13086/Taiwan/2013/KP229376	DuCV/FJzq290/China/2008/GU168779	-	-	-	+	-	+	+
18	3	DuCV/DU092/China/2008/HM162346	DuCV/JSPX03E/China/2016/MF627688	DuCV/VNUA-TB61/Vietnam/2021/ OM176554	-	-	-	+	-	+	+

Table S4. Amino acid variations in ORF2 between 23 DuCV-1b strains and D11-JW-008 within predicted B-cell epitopes

NO.	Strain	Epitopes															
		A	B		C		D	E					/			F	/
		5	56	82	106	107	134	175	183	190	194	195	197	205	213	235	236
1	D11-JW-008/Korea/2011/JQ740363	T	Q	R	S	T	Q	S	I	R	G	T	Y	R	A	V	N
2	D14-RDA-001/Korea/2014/PP056131	.	.	Q	.	K	.	.	V	G	T	.	.	.	T	.	D
3	D14-RDA-003/Korea/2014/PP056132	.	.	.	.	.	.	.	.	G	.	.	.	.	.	.	.
4	D14-RDA-008/Korea/2014/PP056137	.	.	Q	.	K	.	.	.	G	.	.	H	K	T	.	.
5	D14-JW-004/Korea/2014/PP056133	.	.	.	.	.	.	.	.	G	.	.	H	K	T	.	.
6	D14-JW-005/Korea/2014/PP056134	.	.	.	.	.	.	.	.	G	.	.	H	K	T	.	.
7	D14-JW-006/Korea/2014/PP056135	.	.	.	.	.	.	.	.	G	.	.	H	K	T	.	.
8	D14-JW-007/Korea/2014/PP056136	.	.	.	.	.	.	.	.	G	.	.	.	.	.	.	.
9	D14-JW-030/Korea/2014/PP056138	.	.	Q	.	K	.	.	V	G	T	.	.	.	T	.	D
10	D14-JW-031/Korea/2014/PP056139	.	.	Q	.	K	K	.	V	G	S	.	.	.	T	.	D
11	D14-JW-032/Korea/2014/PP056140	.	.	Q	.	K	.	.	V	G	T	.	.	.	T	.	D
12	D14-JW-034/Korea/2014/PP056141	.	.	.	.	.	.	.	.	G	.	.	.	.	.	.	.
13	D14-JW-036/Korea/2014/PP056142	.	T	.	.	.	.	.	.	G	.	.	H	K	T	.	.
14	D14-JW-038/Korea/2014/PP056143	.	.	Q	.	K	.	.	V	G	T	.	.	.	T	.	D
15	D14-JW-039/Korea/2014/PP056144	.	.	.	.	.	.	.	.	G	.	.	H	K	T	.	.
16	D14-JW-040/Korea/2014/PP056145	.	.	Q	.	K	.	A	V	G	T	.	.	.	T	.	D
17	D15-MR-122/Korea/2015/PP056147	.	.	Q	.	K	K	.	V	G	S	.	.	.	T	.	D
18	D15-KW-138/Korea/2015/PP056146	.	.	Q	.	K	.	.	V	G	S	.	.	.	T	.	.
19	D16-KW-035/Korea/2016/PP056148	.	.	Q	.	K	.	.	V	G	S	.	.	.	T	.	.
20	D19-MR-010/Korea/2019/PP056151	.	.	Q	.	K	.	.	V	G	T	.	.	.	T	.	D
21	D19-ETC-001//Korea/2019/PP056150	.	.	Q	.	K	.	.	V	G	T	.	.	.	T	.	D
22	D20-MR-006/Korea/2020/PP056152	.	.	Q	.	K	.	A	V	G	T	.	.	.	T	M	D
23	D22-JW-002/Korea/2022/PP056153	.	.	Q	.	K	.	.	V	G	T	.	.	.	T	.	D

24	D22-MR-002/Korea/2022/PP056154	.	.	.	.	.	.	.	.	G	.	.	H	K	T	.	D
